# Phase-Selective Epitaxy of Trigonal and Orthorhombic Bismuth Thin Films on Si (111)

**DOI:** 10.3390/nano13142143

**Published:** 2023-07-24

**Authors:** Abdur Rehman Jalil, Xiao Hou, Peter Schüffelgen, Jin Hee Bae, Elmar Neumann, Gregor Mussler, Lukasz Plucinski, Detlev Grützmacher

**Affiliations:** 1 Peter Grünberg Institute (PGI-9), Forschungszentrum Jülich, 52425 Jülich, Germany; 2 JARA-FIT (Fundamentals of Future Information Technology), Jülich-Aachen Research Alliance, Forschungszentrum Jülich and RWTH Aachen University, 52425 Jülich, Germany; 3 Peter Grünberg Institute (PGI-10), JARA-Green IT, Forschungszentrum Jülich, 52425 Jülich, Germany; 4 Peter Grünberg Institute (PGI-6), Forschungszentrum Jülich, 52425 Jülich, Germany; 5 Helmholtz Nano Facility (HNF), Forschungszentrum Jülich, 52425 Jülich, Germany

**Keywords:** topological thin films, phase-change materials, structural transformation, phase-selective growth, molecular beam epitaxy, neuromorphic platform

## Abstract

Over the past three decades, the growth of Bi thin films has been extensively explored due to their potential applications in various fields such as thermoelectrics, ferroelectrics, and recently for topological and neuromorphic applications, too. Despite significant research efforts in these areas, achieving reliable and controllable growth of high-quality Bi thin-film allotropes has remained a challenge. Previous studies have reported the growth of trigonal and orthorhombic phases on various substrates yielding low-quality epilayers characterized by surface morphology. In this study, we present a systematic growth investigation, enabling the high-quality growth of Bi epilayers on Bi-terminated Si (111) 1 × 1 surfaces using molecular beam epitaxy. Our work yields a phase map that demonstrates the realization of trigonal, orthorhombic, and pseudocubic thin-film allotropes of Bi. In-depth characterization through X-ray diffraction (XRD) techniques and scanning transmission electron microscopy (STEM) analysis provides a comprehensive understanding of phase segregation, phase stability, phase transformation, and phase-dependent thickness limitations in various Bi thin-film allotropes. Our study provides recipes for the realization of high-quality Bi thin films with desired phases, offering opportunities for the scalable refinement of Bi into quantum and neuromorphic devices and for revisiting technological proposals for this versatile material platform from the past 30 years.

## 1. Introduction

**Figure 1:**
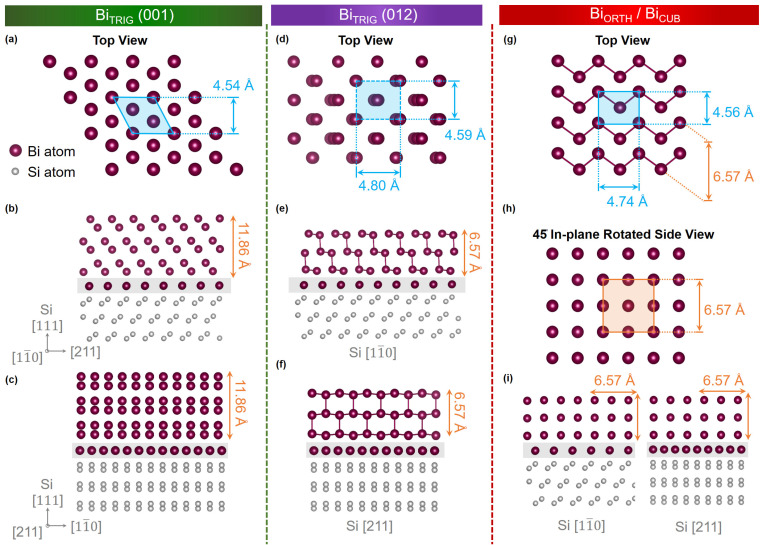
Crystal structure illustrations of Bismuth nanofilm allotropes. (**a**) Top surface view of Bi_TRIG_ (001) where the blue region highlights the 2D in-plane lattices, cross-sectional side view of one unit-cell thick epilayer of Bi_TRIG_ (001) along (**b**) Si $\left[1\bar{1}0\right]$ and (**c**) Si [211] projections. (**d**) Top surface view of Bi_TRIG_ (012) orientation having two monolayer thick film, cross-sectional side view of one unit-cell thick epilayer of Bi_TRIG_ (012) along (**e**) Si $\left[1\bar{1}0\right]$ and (**f**) Si [211] projections. (**g**) Top surface view of Bi_ORTH_ phase, (**h**) side view at 45 rotated plane, (**i**) cross-sectional side view of one unit-cell thick epilayer of Bi_ORTH_ (010) along Si $\left[1\bar{1}0\right]$ and Si [211] projections.

Bi is the heaviest (Z = 83) non-radioactive element that exhibits strong spin-orbit coupling [1,2] and as such, provides a unique and extremely versatile material platform. The strong spin-orbit coupling renders many Bi modifications topologically non-trivial [3,4,5,6], including higher-order topological insulator phase [7,8]. The thinnest of all topologically non-trivial modifications is Bismuthene [9,10]. Analogous to graphene, Bismuthene consists of a monolayer of Bi in a honeycomb lattice. This monolayer has been shown to be a quantum-spin-Hall (QSH) insulator with helical edge states and an inverted bandgap as large as 800 meV. According to the literature, not only Bismuthene, but also thin Bi layers (<1 nm) of trigonal (Bi_TRIG_) and orthorhombic (Bi_ORTH_) phases (Figure 1) exhibit a QSH phase, rendering them 2D topological insulators, too [11,12,13,14]. The difficulty of creating coalesced, phase-pure thin films of homogenous thickness below 1 nm, however, makes it impossible to provide the desired crystals for exploiting Bi-based 2D TIs for technological purposes in the field of quantum spintronics and topological quantum computation.

While Bismuthene comes with its own challenges, such as its restriction to SiC substrates, Bi_ORTH_ and Bi_TRIG_ have been successfully grown on top of HOPG [13,15], sapphire [16], InP (111) [14], glass [17], mica [18], and extensively on Si (111). While growing on Si (111) substrates, all reported growths were conducted on either $\sqrt{3}\times \sqrt{3}-\beta$ [19,20] or 7×7 [21,22,23,24,25] reconstructed surfaces. Both reconstructions facilitate epitaxy in the early stages of growth by providing a high density of nucleation sites; however, both result in an increased number of rotational domains with relatively small grain sizes, reducing the overall quality of epilayers [26,27,28]. Additionally, the maximum height before leaving the topological phase, approx. 0.8 nm for Bi_TRIG_ and 1.3 nm for Bi_ORTH_ phases, limits the realization of high-quality coalesced thin films in the 2D TI regime. Interestingly, the critical value of 1.3 nm in the Bi_ORTH_ phase is related to the amount of buckling in the epilayer. Hence, by finding a way to reduce the buckling, even thicker layers of Bi_ORTH_ might transform into the topological regime, opening an avenue to grow high-quality fully coalesced topological Bi thin films. As found in this study, the growth on Si (111) 1×1 offers unique possibilities in this regard.

Here, a systematic growth study of Bi epilayers, via molecular beam epitaxy on Si (111) 1×1− Bi terminated surfaces was performed. The advantages of opting for the Si (111) 1×1 surface over 7×7 and $\sqrt{3}\times \sqrt{3}-\beta$ reconstructed surfaces are exemplified by the successful achievement of phase-pure epitaxy of Bi_TRIG_ (001) and Bi_ORTH_ (010) thin films with thicknesses up to 30 nm. Strikingly, the buckling in the orthorhombic phase on 1×1 reconstruction is highly reduced compared to 7×7 and$\;\sqrt{3}\times \sqrt{3}-\beta$, which moves the critical value for a cross-over toward the topologically trivial regime to a thickness of 9 nm.

Using systematic selection of growth parameters, a phase map is introduced where the optimum growth parameters for Bi_TRIG_ (001), Bi_TRIG_ (012) and Bi_ORTH_ (010) allotropes are identified. Previous studies have encountered challenges in distinguishing between different nanofilm allotropes of bismuth, leading to uncertainties in characterization. Thin films of some phases exhibit almost identical top surfaces, whereas they significantly differ in the in-plane structure. An overview of crystal structure, depicting the top and cross-sectional side-facets of all attained allotropes in this work, is presented in Figure 1, where the similarity between Bi_TRIG_ (012) and Bi_ORTH_ top-surfaces cannot be ignored. As most of the reported characterizations are based on top-surface investigations, including STM/STS [13,16,20,21,29], AFM [15] and LEED [22,27], the differentiation between various phases is challenging and somewhat vague. By conducting in-depth in-plane structural investigations on both single- and polycrystalline epilayers, this study offers a comprehensive understanding of domain alignment in different phases, effectively resolving any ambiguity in distinguishing between Bi_TRIG_ (012) and Bi_ORTH_ phases. Taking earlier reports on Bi nanofilm allotropes into account, several unsolved questions regarding the possibility of phase segregation, phase stability, phase transformation, and phase-dependent thickness limitations are systematically exploited and addressed in this study.

In addition to the opportunities this study creates for exploring topological devices, the optimized growth techniques developed in this research hold the potential to benefit a wide range of other fields as well. Topologically trivial Bi undergoes an electronic transition from a semimetal to a semiconductor through quantum confinement [30,31,32]. In its semiconducting regime, Bi can be exploited in quantum sensors [33] or used as a ferroelectric [15]. Additionally, due to its poor thermal stability, Bi is a suitable candidate for phase-change applications where the crystalline Bi film can be transformed into an amorphous state with relatively low applied energy [34]. The thermal instability of Bi_TRIG_ and ferroelectric attributes of Bi_ORTH_ designate Bi, in either phase, as one of the most promising candidates for low-power neuromorphic applications. Overall, we are confident that the results of this study will provide the respective communities with significant insight into how to prepare high-quality and phase-segregated crystalline epilayers, so that Bi in the required phase can be readily incorporated into the respective device platforms in a scalable manner.

## 2. Growth Optimization and Characterization of Bi_TRIG_

Single-elemental thin films entail an immensely diverse temperature range to crystalize in certain phases. Some elements demand ultra-low temperatures (below 0 °C) to crystallize, such as Al and Sn [35,36], while others require temperatures as high as 1400 °C, such as Ta. Bi, however, tends to crystallize at room temperature. In order to grow smooth and high-crystal-quality films of Bi via MBE, the optimum growth parameters, i.e., the growth temperature (T_sub_) and the thin-film growth rate (R_TF_), must be identified.

**Figure 2:**
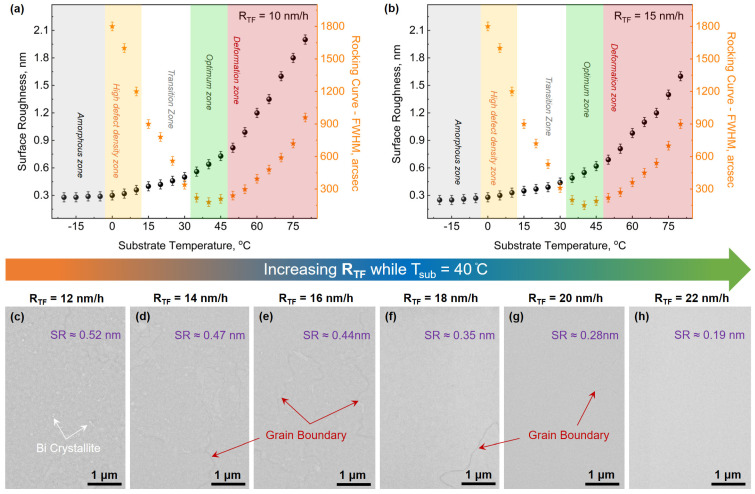
Identification of optimum growth parameters of the Bi_TRIG_ (001) phase. Temperature-dependent changes in surface and crystal quality of Bi_TRIG_ (001) epilayers from − 20 °C to 80 °C prepared with (**a**) R_TF_ = 10 nm/h, (**b**) R_TF_ = 15 nm/h. SEM images of Bi_TRIG_ (001) epilayers prepared at T_sub_ = 40 °C, while R_TF_ is varied from (**c**) 12 nm/h, (**d**) 14 nm/h, (**e**) 16 nm/h, (**f**) 18 nm/h, (**g**) 20 nm/h and (**h**) 22 nm/h, confirming the improved surface quality. SR stands for “surface roughness”.

Initially, to acquire the Bi_TRIG_ (001) phase, the growth of Bi films is conducted over a T_sub_ range from −20 °C to 80 °C, where the temperature of the Bi effusion cell is adjusted to maintain R_TF_ = 10 nm/h. The morphology of the films is analyzed via scanning electron microscopy (SEM), while the structural characterizations are performed via XRD. The X-ray reflectometry (XRR) scans have provided the necessary information about the thickness and roughness of each film. It is observed that films prepared at low temperatures exhibit smoother surfaces, with roughness increasing significantly with increasing T_sub_, as can be seen in Figure 2a. Preliminary XRD investigations have revealed diverse structural behavior of the Bi films, including amorphous, single-, and polycrystalline states over the entire range of T_sub_. The results of qualitative investigations of Bi_TRIG_ (001) crystals, acquired via rocking-curve (RC) analysis of the Bi_TRIG_ (0003) peak, can be visualized in Figure 2a. A similar set of growths is conducted with an increased R_TF_ = 15 nm/h, and while a similar trend is observed, there appears to be a slight improvement in surface and crystal quality. The summarized results of this R_TF_ can be visualized in Figure 2b.

Based on RC analysis, the investigated temperature range is divided into distinct categories that are named “amorphous”, “high defect density”, “transition”, “optimum” and “deformation” zones. The corresponding temperature zones are highlighted in grey, orange, white, green and red, respectively, in Figure 2a,b. The Bi films prepared between −20 °C and 0 °C are observed to be either amorphous or partially crystalline and are thus placed in the “amorphous zone”. The films prepared between 0 °C and 15 °C are witnessed to be crystalline in the Bi_TRIG_ (001) phase; however, these films exhibit a poor crystal quality with RC full-width-half-maxima (FWHM) values above 900″. This temperature range is thus categorized as the “high defect-density zone”. The average grain size and henceforth, the crystal quality is observed to improve continuously throughout the “transition zone” between 15 °C and 35 °C, with the RC values reaching close to 300″. The films prepared in the “optimum zone” with T_sub_ ranging between 35 °C and 50 °C are observed to have the best crystal quality where RC values below 200″ are achieved. Above 50 °C, epilayers are observed to degrade in structural quality, as seen by the increase in RC, and the crystal is observed to transform into a polycrystalline state. This occurs due to an increased density of point and structural defects. This increase in defect density arises as the epilayer becomes more and more thermally unstable with increasing T_sub_. This temperature range is thus categorized as the “deformation zone”. With the identification of the “optimum zone”, T_sub_ = 40 °C is witnessed to deliver the best results and, therefore, is selected for all future growths of the Bi_TRIG_ (001) phase. The focus is then shifted to the identification of optimum R_TF_. As witnessed in Figure 2a,b, compared to R_TF_ = 10 nm/h, both features, i.e., the surface and crystal quality, are observed to improve at R_TF_ = 15 nm/h. It points toward a higher probability of achieving optimum growths at R_TF_ > 15 nm/h. Following that, several Bi_TRIG_ (001) epilayers are prepared where R_TF_ is gradually (in steps of 2 nm/h) increased from 10 nm/h to 30 nm/h, while T_sub_ is kept constant at 40 °C. The corresponding changes in the surface morphology can be observed via SEM images. A few images, corresponding to key R_TF_ values, are depicted in Figure 2c–h. Through the SEM images, a substantial improvement in the surface quality of the epilayers is observed with increasing R_TF_. Not only do the Bi crystallites seen at lower R_TF_ vanish, but the averaged grain size is also observed to enlarge significantly. Though epilayers are prepared with R_TF_ up to 30 nm/h, the best results are obtained at R_TF_ = 22 nm/h, and thus, this is selected as the optimum R_TF_.

**Figure 3:**
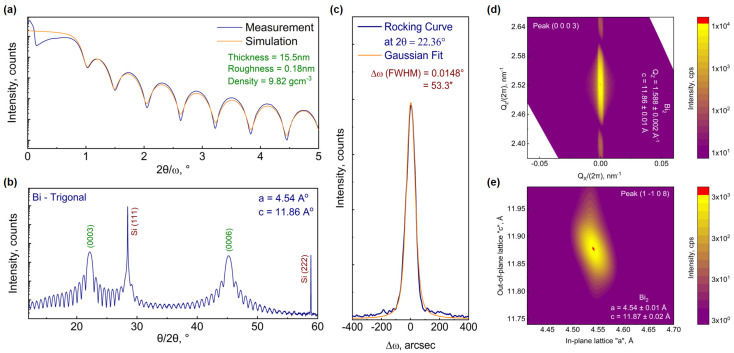
XRD characterization of Bi_TRIG_ (001) epilayer prepared at the optimal growth parameters. (**a**) Measured and simulated XRR curves providing information about the thickness and roughness of the epilayer, (**b**) XRD θ/2θ scan confirming the single crystallinity, (**c**) rocking curve acquired at Bi_TRIG_ (0003) peak, (**d**) symmetric RSM across Bi_TRIG_ $\left(0003\right)$ peak to evaluate the out-of-plane lattice parameter and to identify the presence of any tilted grains and (**e**) asymmetric RSM across Bi_TRIG_ $\left(1\bar{1}08\right)$ peak to evaluate the in-plane lattice parameters.

With the optimal growth parameters (T_sub_, R_TF_) = (40 °C, 22 nm/h) for the Bi_TRIG_ (001) phase found, several epilayers with various thicknesses are prepared to confirm the growth reproducibility by analyzing the surface roughness and crystal quality of each epilayer. After confirming reproducible growths, detailed structural characterizations are performed via XRD, and the lattice parameters of Bi_TRIG_ epilayers are evaluated. Figure 3a represents the measured XRR and simulated curves of a 16 nm thick epilayer. The fitting parameters confirm the ultra-smooth surface, where the measured roughness is even better than the Si (111) substrate itself, which is not something one would expect in MBE. The reason for achieving such an ultra-smooth epilayer resides purely in the structural arrangement of Bi_TRIG_. As introduced in Figure 1, the Bi_TRIG_ (001) crystals are arranged in vertically stacked bilayers where the height of a single bilayer (3.9 Å) matches almost perfectly with the Si step-edge height (3.87 Å) [5,6]. It is known that Si step edges act as nucleation sites in an early stage of growth [37]. However, during the growth of layered materials such as Bi_2_Te_3_ and Sb_2_Te_3_, these step heights lead to the formation of extended structural defects, including antiphase domains and screw dislocations [5,6], whereas, in the case of Bi_TRIG_, a bilayer with matching height inserts itself at the step edge, neutralizes the formation of extended defects, and results in an improved crystal and surface quality of the epilayer. Figure 3b represents an XRD θ/2θ scan confirming the single crystalline behavior of the Bi_TRIG_ (001) epilayer. It is also due to the acquired ultra-smooth surface that the thickness oscillations are observed to appear all the way up to 60° in the diffraction pattern.

Ensuring the single crystallinity of the epilayer, the qualitative investigations are performed via RC (Δω) analysis at the Bi_TRIG_ (0003) peak. Figure 3c depicts the measured RC and the corresponding Gaussian fit, providing the FWHM value of 53 ± 10″. To the best of our knowledge, such a low FWHM value in the MBE-grown Bi-based materials has never been reported. Finally, the lattice parameters are evaluated using the symmetric and asymmetric reciprocal space maps (RSMs). Figure 3d represents a symmetric map across the Bi_TRIG_ (0003) peak. The measured value provided $Q_{Z}$ = 2π (2.529) ± 0.002 nm^−1^ = 1.588 ± 0.002 Å^−1^ which results in an out-of-plane lattice “c” = 11.86 ± 0.01 Å. This acquired value matches perfectly with the reported value in the literature, as mentioned in Figure 1a. It is worth mentioning here that the acquired $Q_{X}$ value is zero, and thus, any information about the in-plane lattice cannot be extracted. In symmetric setting, x-rays probe only the vertical lattice and provide high-precision information only about the out-of-plane parameter. It, however, lacks any information about the in-plane structure. Depending on material composition and crystal symmetry, XRD peaks differ in their intensity. Considering the Bi_TRIG_ (001) phase, the asymmetric $\left(1\bar{1}08\right)$ peak is selected to probe the in-plane structure, and the lattice information is extracted. The corresponding RSM is depicted in Figure 3e. As both in-plane lattices are identical (trigonal structure), a single asymmetric peak is sufficient to evaluate the lattice information.

## 3. Structural Transformation and Phase Map

With the successful growth of Bi_TRIG_ (001) epilayers, the focus is shifted to the earlier introduced “deformation zone”. As stated in Figure 2, the crystal is observed to exhibit a polycrystalline state when exposed to T_sub_ > 50 °C. Preliminary XRD investigations indicated that it is either the Bi_TRIG_ (012) or the Bi_ORTH_ phase (as both phases exhibit identical out-of-plane lattices), co-existing with the Bi_TRIG_ (001). The existence of Bi_ORTH_ phase has already been reported in the literature, however, only in the form of non-coalescent ultra-thin (<2 nm) crystallites [21,38,39]. A recent study has reported the presence of 60-nm-wide Bi_ORTH_ crystal grains reaching the thickness of approx. 25 nm in polycrystalline films [26]. Thus, the possibility of growing thick Bi_ORTH_ crystallites is addressed, while the uncertainty of achieving phase-pure, fully coalesced, and high-crystal-quality Bi_ORTH_ epilayers still remains. These obscurities must be tackled before the incorporation of Bi_ORTH_ in nano-devices becomes a reality.

**Figure 4:**
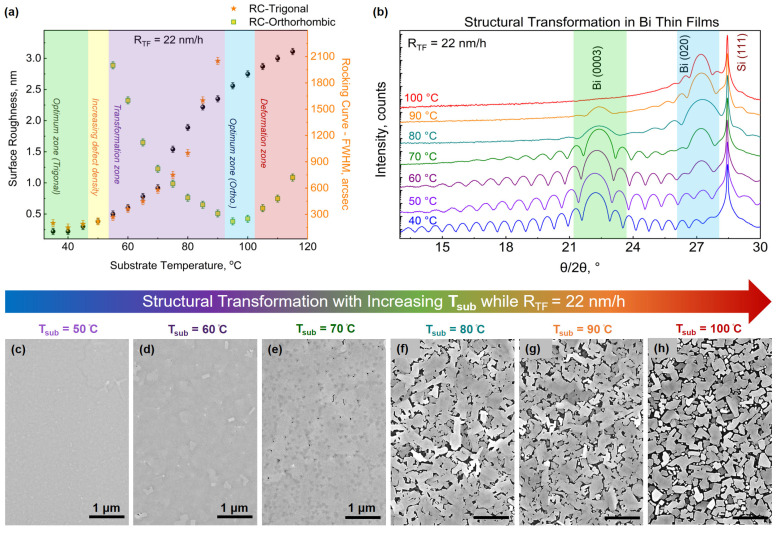
Structural transformation in Bi epilayers. (**a**) Temperature-dependent changes in surface roughness (indicated by black data points) and crystal quality of Bi epilayers from 40 °C to 120 °C, prepared with R_TF_ = 22 nm/h, result in continually changing structures from Bi_TRIG_ (001) to Bi_ORTH_ (010) phase passing through polycrystalline states. (**b**) The corresponding diffraction pattern at the select key temperatures indicating the continually reducing and enhancing contributions from Bi_TRIG_ (001) and Bi_ORTH_ (010) phases, respectively. For better understanding, Bi_TRIG_ (0003) and Bi_ORTH_ (020) peak regions are highlighted with green and blue, respectively. The corresponding changes in surface morphology via SEM images when the epilayer is prepared at (**c**) T_sub_ = 50 °C, (**d**) 60 °C, (**e**) 70 °C, (**f**) 80 °C, (**g**) 90 °C, and (**h**) 100 °C.

Considering the significance of Bi_ORTH_ to neuromorphic, ferroelectric, and topological communities, a systematic search for the Bi_ORTH_ phase is initiated. Keeping R_TF_ at the optimum value of 22 nm/h, adopted for the Bi_TRIG_ (001) phase, a series of growths are conducted where T_sub_ is gradually (in steps of 2 °C) increased from 40 °C to 120 °C. The structural investigations are performed, and a summary of the acquired results is plotted in Figure 4a. Figure 4b represents the diffraction pattern at select key data points, and Figure 4c–h depict the corresponding morphological alterations via SEM images. At low temperatures, the surface roughness is observed to increase gradually until T_sub_ reaches 70 °C, after which a rapid increment in the surface roughness is evident. This behavior can also be witnessed in Figure 4b with the vanishing thickness oscillations in the corresponding diffraction patterns. The crystal quality of the Bi_TRIG_ (001) phase is observed to continually degrade with increasing T_sub_, as can be seen with the increasing FWHM values in Figure 4a. After T_sub_ = 55 °C, the Bi_ORTH_ phase started to emerge, initially with a poor crystal quality that continually improved with increasing T_sub_ until 105 °C. For better understanding, the earlier designated “deformation zone” in Figure 2 is dissected into further sub-groups. In the region from T_sub_ = 55 °C to 95 °C, only polycrystalline layers (containing both the Bi_TRIG_ (001) and the Bi_ORTH_ phases) are achieved. Due to the continually decreasing fraction of Bi_TRIG_ (001) and increasing ratio of Bi_ORTH,_ this region is named the “transformation zone”. Above the transformation zone, a small set of temperatures, i.e., 95–105 °C, have provided the pure phase of the Bi_ORTH_ crystals. Though the surface quality of acquired Bi_ORTH_ is far from optimal, the crystal quality is at an acceptable level, with the FWHM value of the Bi_ORTH_ (020) peak reaching below 300″. This region is designated the “optimum zone (orthorhombic)”. Above this temperature, the crystal is only observed to degrade, thus starting the actual “deformation zone”.

Before the focus is shifted to optimizing the surface quality of Bi_ORTH_ crystals, a couple of points are worth mentioning here. (1) For the first time, phase-pure growth of Bi_ORTH_ crystals having a thickness >> 2 nm is successfully achieved via MBE. (2) Though Bi_ORTH_ peaks started to emerge at T_sub_ > 55 °C, a sudden change in crystal morphology and structural alignment is observed to transpire in the transformation zone between 70 °C and 80 °C. Referring to Figure 4e,f, the presence of a relatively smooth and almost fully coalesced layer at 70 °C is suddenly converted into rough and randomly ordered crystals at 80 °C, confirming a sudden rather than a gradual change in the crystal structure. Moreover, notice the change in XRD pattern between 70 °C and 80 °C in Figure 4b, where a clear indication of contributing layer thickness change can be perceived. The preliminary characterizations of the in-plane structure indicate that from 55 °C to 70 °C, the peak appearing at 2θ = 27.2° belongs to the Bi_TRIG_ (012) phase, and not the Bi_ORTH_. Above 70 °C, however, the crystal re-arranges itself, undergoes a few transformations, and converts into the Bi_ORTH_ phase that slightly differs from the Bi_TRIG_ (012) orientation. It is interesting to note that the out-of-plane lattices in Bi_TRIG_ (012) and Bi_ORTH_ match perfectly, and this is the reason that no significant shift in the diffraction peak is observed. At this stage, it can be assumed that the Bi_TRIG_ (012) orientation acts as an intermediate stage between Bi_TRIG_ (001) and Bi_ORTH_ phases. Further discussions on similarities and differences between both phases, i.e., the Bi_TRIG_ (012) and the Bi_ORTH_, will be conducted at a later stage; however, right now the focus will be kept on the surface optimization of the Bi_ORTH_ phase.

**Figure 5:**
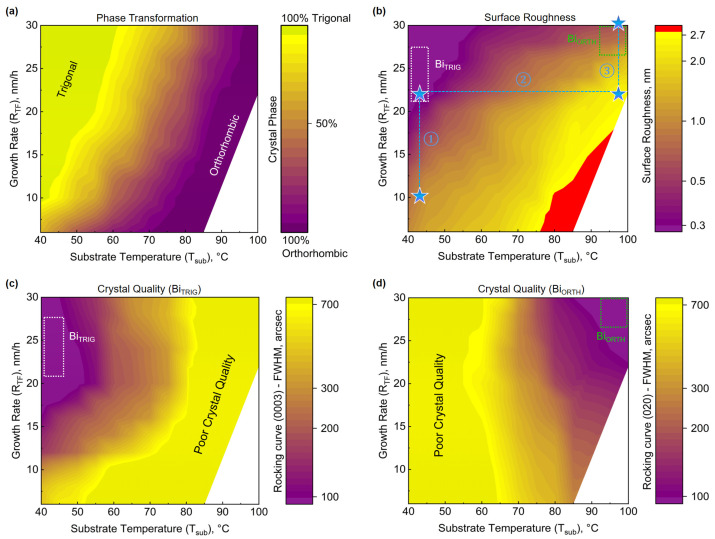
Growth parameter-dependent structural transformation map. A summary of 445 data points where the growths are performed between the two distinct optimum zones, i.e., trigonal and orthorhombic phases. XRD evaluations are abridged in colored maps providing the information about (**a**) the relative presence of certain allotropes between the Bi_TRIG_ (001) and the Bi_ORTH_ (010) phases in the epilayers, (**b**) the surface roughness of the epilayers at the corresponding data points. The red color in the map indicates the rms roughness exceeds 3 nm. The path adopted during initial investigations of the structural optimization and the phase transformation in Bi thin-films is highlighted with blue dotted lines, where, the stars represent key data points. The sub-paths ① and ③ represent R_TF_ dependent surface optimization of the Bi_TRIG_ (001) and the Bi_ORTH_ (010), respectively while ② represents the temperature dependent phase transformation in Bi thin films with R_TF_ is kept at 22 nm/h. The qualitative figure of merit for the structural quality of the trigonal and the orthorhombic phases, at the corresponding data points on the map, are depicted in (**c**) and (**d**) by measuring the rocking curves at Bi_TRIG_ (0003) and Bi_ORTH_ (020) peaks, respectively.

Based on the analysis of Figure 4, it is clear that the Bi_ORTH_ phase can only be segregated at high temperatures (around 100 °C). What role R_TF_ plays in the phase segregation of Bi films is still unknown. Furthermore, it is also important to study the impact of R_TF_ on surface quality to achieve smooth and fully coalesced Bi_ORTH_ epilayers if possible. To answer these questions, a detailed and systematic growth study of Bi films is conducted where T_sub_ is altered in steps of ±2 °C between the two identified “optimum zones” depicted in Figure 4a. R_TF_ is also adjusted in steps of 2 nm/h, starting from 6 nm/h to 30 nm/h. All of the prepared films are investigated via XRD, and the information about crystal phase, crystal quality, and surface roughness is evaluated and depicted with the help of colored maps in Figure 5.

The impact of growth parameters (T_sub_, R_TF_) on the crystal phase can be visualized in Figure 5a. To identify the presence of a single phase in the epilayer is straightforward; however, the question arises as to how to distinguish the relative presence of a specific phase in the polycrystalline films. To understand the impact of changing growth parameters, phase-specific diffraction peaks, i.e., Bi_TRIG_ (0003) and Bi_ORTH_ (020), are selected. The measured peak intensities in the diffraction pattern, belonging to a specific film thickness, are multiplied with the corresponding theoretically predicted intensities. The ratio $R_{\left(0003\right)}/R_{\left(020\right)}$ is evaluated and utilized to identify the relative presence of a certain phase in the epilayer. Similarly, the corresponding information at each data point about the surface roughness and crystal quality, RC-FWHM, for Bi_TRIG_ (0003) and Bi_ORTH_ (020) peaks is represented in Figure 5b–d, respectively.

It can be clearly observed that low T_sub_ and high R_TF_ facilitate the achievement of the Bi_TRIG_ (001) phase. Conversely, at high T_sub_ and low R_TF_, films crystallize only in the Bi_ORTH_ phase. However, none of the above-mentioned parameters are optimal. For Bi_TRIG_ (001), high R_TF_ results in smoother films, as can be witnessed in Figure 5b; however, after a certain point, the crystal quality starts to degrade with increasing density of rotational domains and reduced grain size, resulting in enhanced RC-FWHM values that can be visualized in Figure 5c. The best crystal quality is achieved at R_TF_ between 22 and 26 nm/h. The optimum growth zone for Bi_TRIG_ (001) is highlighted with the white-dotted box in Figure 5b,c. As far as the Bi_ORTH_ phase is concerned, films achieved at low R_TF_ values exhibit enormously high surface roughness due to the formation of non-coalescing crystallites. Such films are not suitable for integration into any electronic device. With increasing R_TF_, the surface roughness is witnessed to improve significantly; however, the temperature requirements to keep the Bi_ORTH_ phase segregated increase as well. Epilayers with the best crystal quality and improved surfaces of the Bi_ORTH_ phase are achieved at high T_sub_ and R_TF_ values, a region marked with the green-dotted box in Figure 5b,d.

Observing the phase transformation map, it is evident that almost 80% of the data points resulted in the formation of polycrystalline films. It emphasizes the importance of identifying phase-specific optimum growth zones. Moreover, the achievement of pure-phase and thick crystalline films of the Bi_ORTH_ phase has not been reported to date. Thus, the transformation maps depicted in Figure 5 not only helped in achieving phase-pure epilayers of Bi_ORTH_ systematically, but also provided a platform that serves as a reference for all future requirements of phase-segregated growths of Bi nanofilm allotropes. To highlight the importance of these maps, the path of parameter selection in this study is retraced in Figure 5b, which leads to the next step, i.e., the surface optimization of Bi_ORTH_ films.

## 4. Growth Optimization of Bi_ORTH_

**Figure 6:**
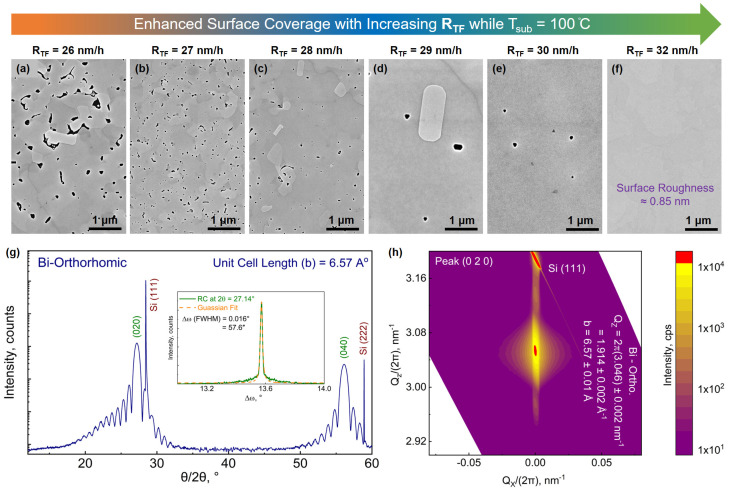
Growth optimization and structural characterization of Bi_ORTH_ epilayers. SEM images represent continually improving surface coverage when the epilayers are prepared with (**a**) R_TF_ = 26 nm/h, (**b**) 27 nm/h, (**c**) 28 nm/h, (**d**) 29 nm/h, (**e**) 30 nm/h and (**f**) 32 nm/h while T_sub_ is kept at 100 °C. (**g**) Diffraction pattern of Bi_ORTH_ epilayer prepared at 32 nm/h, (inset) rocking curve acquired at Bi_ORTH_ (020) peak, (**h**) symmetric RSM acquired at Bi_ORTH_ (020) peak, providing information about the out-of-plane lattice parameters and confirming high crystal quality of the Bi_ORTH_ epilayer.

As depicted in Figure 5b, with an increment in R_TF_, the surface roughness of Bi_ORTH_ epilayers improves considerably. By further increasing R_TF_ to 32 nm/h, almost fully coalesced films are successfully achieved. Though random small holes are still observed in the films, a majority of the surface is observed to be fully coalesced. The corresponding changes in morphology can be visualized with the help of SEM images in Figure 6a–f.

Later, XRD-based structural characterizations are performed, and the diffraction pattern of the best acquired film at R_TF_ = 32 nm/h is plotted in Figure 6g. The appearance of the thickness oscillations in Figure 6g, compared to one depicted in Figure 4b at T_sub_ = 100 °C and R_TF_ = 22 nm/h, is an indication of significant improvement in the surface quality. The RC measurement at the Bi_ORTH_ (020) peak, depicted as inset in Figure 6g, has provided the FWHM value of 58 ± 10″, which is comparable to the crystal quality of the Bi_TRIG_ (001), depicted in Figure 3c. To evaluate the out-of-plane lattice parameters and to identify the presence of any tilted grains, a symmetric RSM across the Bi_ORTH_ (020) peak is acquired and depicted in Figure 6h. The measured value provided $Q_{Z}$ = 2π × (3.046) ± 0.002 nm^−1^ = 1.914 ± 0.002 Å^−1^, which results in an out-of-plane lattice “b” = 6.57 ± 0.01 Å. The acquired value is in good agreement with earlier reported results on Si (111) substrates [21,26,27]. The symmetric RSM also confirms the absence of any tilted or heavily misaligned grains, pointing to a high-quality epilayer. As discussed earlier, to extract the in-plane lattice parameters, asymmetric diffraction data must be attained. However, before any asymmetric measurements can be performed, it is important to gain a better understanding of how the domains prefer to align with respect to the substrate.

## 5. Domain Alignment of Bi Nanofilm Allotropes

**Figure 7:**
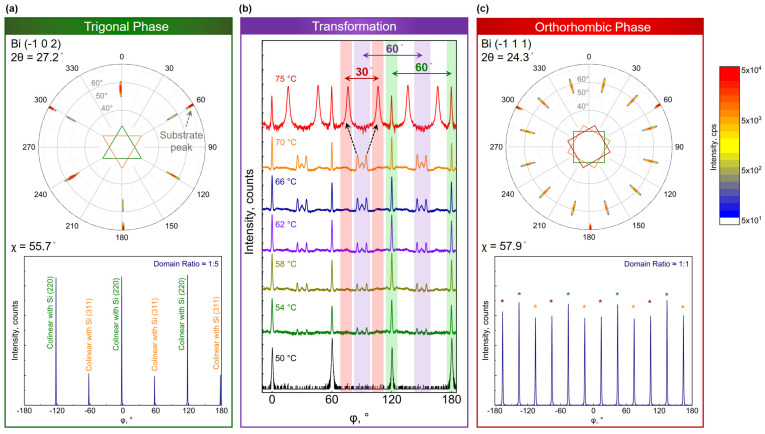
Domain analysis of Bi nanofilm allotropes and their alignment with respect to the substrate. (**a**) Analysis of the Bi_TRIG_ (001) epilayers performed by probing the $\left(1\bar{1}02\right)$ peak. (**Top**) A pole-figure map representing six distinct peaks originating from two 60° in-plane rotated trigonal domains. (**Bottom**) The φ -scan, a 2D cut at χ = 55.7°. (**b**) A series of φ -scans representing temperature-dependent structural transformation in Bi epilayers. The emergence of three-pointed distinct peaks is found to be related to the Bi_TRIG_ (012), whereas the transformation of those three-pointed peaks into 12 distinct peaks is an indication of the orthorhombic crystal structure. (**c**) Analysis of the Bi_ORTH_ (010) epilayers conducted via probing $\left(\bar{1}11\right)$ peak. (**Top**) A pole-figure map representing 12 distinct peaks originating from three 30° in-plane rotated orthorhombic domains highlighted with district colored and rotated squares. (**Bottom**) The φ -scan, a 2D cut at χ = 59.9°, provides information about the relative presence of domains in the epilayer. Each peak is marked with a color that corresponds to the associated domain.

To comprehend the in-plane alignment of domains with respect to the substrate, the rotational symmetry of lattices via conventional φ-scanning is investigated. Figure 7 represents detailed information on domain alignment in various Bi phases. A trigonal lattice exhibits 3-fold rotational symmetry. While growing a trigonal crystal on a hexagonal surface, e.g., Si (111), the crystal may exhibit 60° in-plane rotated domains. A similar scenario is observed in Bi_TRIG_ (001) epilayers, as depicted in Figure 7a. The figure on top is a 3D pole-figure map, acquired via probing the Bi_TRIG_ $\left(1\bar{1}02\right)$ peak to identify all present domains in the epilayer, as this peak exhibits the highest diffraction intensity. It is evident that the epilayer contains twin domains, i.e., 60° rotated domains that can also be visualized with the help of an illustration containing two 60° rotated colored triangles placed inside the pole-figure map. Moreover, the domains are observed to be collinear with the substrate peak, referring to the 60°, 180°, and 300° marks in the map. At the bottom of Figure 7a, a 2D cut at *χ* = 55.7° is plotted. From the intensity, it is evident that both rotational domains have not populated the epilayer equally; rather, a 1:5 ratio is observed. Other trigonal crystals, e.g., Bi_2_Te_3_ and Sb_2_Te_3_, are also reported to exhibit a similar trend when grown on Si (111) [5,6]. However, in the case of Bi_TRIG_ (001), a minor difference is perceived. It is observed that the Bi_TRIG_ (001) exhibits a preferential alignment to Si (220), which is a behavior opposite to that of Bi_2_Te_3_ and Sb_2_Te_3_ crystals that favor Si (311) instead. Why Bi_TRIG_ behaves differently from other family compounds is not clear at this point and requires further insight with the support of theoretical modeling. Nevertheless, a similar trend is observed in all Bi_TRIG_ (001) epilayers prepared at T_sub_ from 5 °C to 50 °C, while R_TF_ > 10 nm/h.

During this investigation, it was discovered that for T_sub_ > 50 °C, the crystal enters the “transformation zone” where unique in-plane transitions are observed, as visualized in Figure 7b. The 60° rotated domains of Bi_TRIG_ (001) are highlighted with green-colored regions. Starting from T_sub_ = 54 °C, distinctive three-pointed peaks start to emerge in between Bi_TRIG_ (001) domains. These domains, similar to the Bi_TRIG_ (001), are observed to exhibit 60° periodicity; however, their alignment in between the Bi_TRIG_ peaks placed them at a 30° offset with the substrate collinearity. These domains are highlighted with purple-colored regions in Figure 7b. Alongside the appearance of exotic three-pointed peaks in the φ-scan, a diffraction peak at 2θ = 27.2° is witnessed to emerge, as discussed in Figure 4b, which confirms that these two events are linked. Moreover, as the exotic peaks in the φ-scan exhibit 60° periodicity, they cannot originate from the Bi_ORTH_ phase and must be connected to the Bi_TRIG_ (012) orientation, as the Bi_TRIG_ (012) also exhibits the facilitating diffraction peak at 2θ = 27.2°. This assumption, however, requires further investigation. With increasing T_sub_, the relative intensity of these uniquely oriented peaks is also observed to rise until T_sub_ = 70 °C.

Above 70 °C, another in-plane transition is witnessed where each three-pointed exotic peak is observed to split into two distinct peaks that are 30° apart from each other and ±15° shifted from the position of their origin, highlighted by the black-dotted arrows in Figure 7b. Thus, the newly originated peaks exhibit a 45° offset from the substrate collinearity. This transition gives rise to 12 peaks that exhibit 30° periodicity, along with six peaks originating from the Bi_TRIG_ $\left(001\right),$ as observed in an epilayer prepared with T_sub_ = 75 °C and depicted in Figure 7b. As far as the diffraction pattern is concerned, no significant change in peak positions is observed. The morphology of the crystal, on the other hand, is observed to transform completely, as discussed in Figure 4. As the appearance of 12 distinct peaks with 30° periodicity is a hallmark of cubic-like structures when aligned on a hexagonal surface [40], this second stage of transition can be linked to the rise of the Bi_ORTH_ phase.

With further increase in T_sub_, peaks associated with the Bi_TRIG_ (001) are witnessed to diminish, while 30° periodic Bi_ORTH_ peaks are observed to improve in intensity until T_sub_ reaches 95 °C. Above 95 °C, epilayers are observed to crystallize in the Bi_ORTH_ phase only. Figure 7c depicts a 3D pole-figure map of the Bi_ORTH_ epilayer acquired via probing $\left(\bar{1}11\right)$ peak and a 2D cut at χ = 57.9°, confirming the presence of only 12 in-plane peaks. As the orthorhombic lattice exhibits four-fold rotational symmetry, the alignment of domains in the epilayer is illustrated with the help of three distinct colored boxes rotated by 30° and placed inside the pole-figure map. The four-fold rotational symmetry can also be witnessed in a 2D φ-scan where each peak is color-identified with the associated domain. During the first and second in-plane transitions in Figure 7b at 54 °C and 75 °C, respectively, the non-collinearity of the crystal peak with respect to the substrate was explained. Here, all three Bi_ORTH_ domains can be visualized to exhibit a 15°, 45°, and 75° offset with respect to the substrate peak, respectively.

Through detailed analyses of φ-scans, it can be summarized that from the “optimum zone (trigonal)”, i.e., (T_sub_, R_TF_) = (40 °C, 22 nm/h), to the “optimum zone (orthorhombic)”, i.e., (T_sub_, R_TF_) = (100 °C, 32 nm/h), epilayers go through four major states. (1) All epilayers before entering the “transformation zone” exhibit twin domains with six distinct peaks originating from the Bi_TRIG_ (001) phase. (2) Epilayers prepared at 50 °C < T_sub_ ≤ 70 °C exhibit polycrystalline structures featuring the Bi_TRIG_ (001) along with the Bi_TRIG_ (012) orientation. The exotic three-pointed peaks are found to be related to the Bi_TRIG_ (012) orientation, which is confirmed via asymmetric RSM that can be seen in Supplementary Figure S1. (3) Epilayers prepared at 70 °C < T_sub_ < 95 °C also exhibit polycrystalline structures; however, here, the Bi_TRIG_ (001) and the Bi_ORTH_ (010) phases are realized. As the out-of-plane lattices in the Bi_ORTH_ (010) and the Bi_TRIG_ (012) orientations match with good approximation, no significant change in the diffraction pattern is observed (Figure 4b). The signature 12 peaks of orthorhombic crystal along with six peaks of the Bi_TRIG_ are observed in this region. It is also important to point out here that, for a short temperature range, epilayers are also witnessed to contain all three phases, including Bi_TRIG_ (001), Bi_TRIG_ (012) and Bi_ORTH_ (010). As the temperature range to host these three phases is very narrow, φ-scans are not presented in Figure 7. (4) Finally, the phase-pure Bi_ORTH_ epilayers are acquired at T_sub_ ≥ 95 °C, where only 12 distinct peaks of three rotational domains are observed in the epilayer.

## 6. Evaluation of Lattice Parameters of Bi_ORTH_

**Figure 8:**
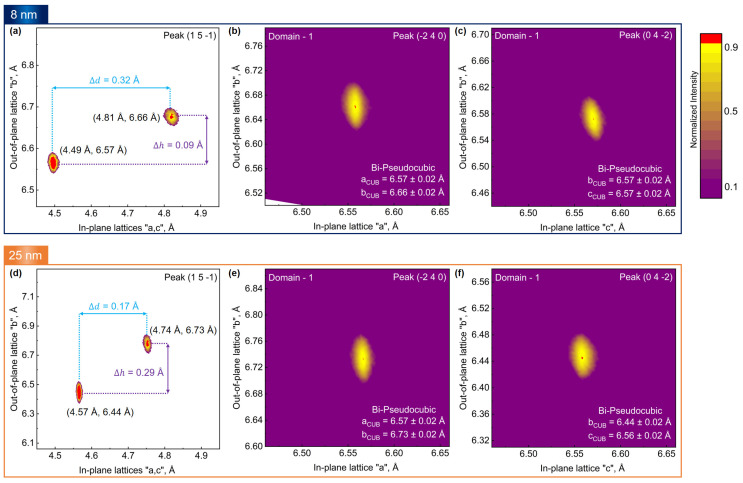
Evaluation of in-plane lattice parameters of the Bi_ORTH_ (010) epilayers via asymmetric RSM. (**a**) A map acquired at the Bi_ORTH_ $\left(15\bar{1}\right)$ peak in an 8.4 nm thick epilayer, where Δd represents the difference in length between in-plane lattices while Δh represents buckling, i.e., high difference between Bi atoms of the same monolayer; the 45° in-plane rotation led to the formation of pseudo-cubic sub-lattice, and the acquired maps at the Bi_CUB_ $\left(\bar{2}40\right)$ and the Bi_CUB_ $\left(04\bar{2}\right)$ peaks are represented in (**b**,**c**), respectively. Similar measurements are performed in a 25 nm thick epilayer at (**d**) Bi_ORTH_$\left(15\bar{1}\right)$, (**e**) Bi_CUB_ $\left(\bar{2}40\right)$ and (**f**) Bi_CUB_ $\left(04\bar{2}\right)$ peaks, where a difference in buckling height in (**d**) is observed to enhance from 0.09 Å to 0.29 Å.

After a thorough understanding of domain alignment in the various Bi phases, the focus is set to evaluate the 2D in-plane lattices of Bi_ORTH_ using asymmetric RSMs. Based on reported Bi_ORTH_ lattices [21,26], the Bi_ORTH_ $\left(06\bar{2}\right)$ and the Bi_ORTH_ (260) peaks are probed (details can be found in Supplementary Figures S2–S4), and unexpectedly, no diffraction is observed. It indicates the selected lattice parameters are incorrect. After several failures to probe individual in-plane lattices, successful diffractions are finally achieved for 45° rotated the Bi_ORTH_ $\left(15\bar{1}\right)$ and the Bi_ORTH_ $\left(\bar{1}71\right)$ peaks, and, as expected (orthorhombic crystal, unlike cubic, exhibits nonequivalent in-plane lattices), dual diffraction peaks are detected. These peaks contain information about both in-plane lattices but are not ideal for extracting lattice information as they have reduced diffraction intensity. Nonetheless, by probing these peaks, the diffraction is successfully witnessed for each 30° rotated domain, as explained in Figure 7c. One of the asymmetric RSMs acquired at the Bi_ORTH_ $\left(15\bar{1}\right)$ peak is depicted in Figure 8a, where the in-plane lattice parameters are found to be 4.49 ± 0.02 Å and 4.81 ± 0.02 Å, respectively. The measured lattices are in close agreement with reported values of Bi_ORTH_ on HOPG [10,13,39], but they differ slightly from reported values acquired on Si (111) [21,26,27,29,41].

Analyzing RSM in Figure 8a, it can be seen that both in-plane lattices are unequal, having a difference of Δd = 0.32 Å. Moreover, a minute difference along the z-direction of both peaks is also evident and is measured to be Δh = 0.09 Å, which can be related to buckling [13,15,29]. Buckling is a strain-induced deformation in a crystal and can be described as the height difference between two atoms of the same monolayer [13]. During the lattice transformation of Bi_TRIG_ (012), a phase known to exhibit strong buckling, into Bi_ORTH_, the strain relaxation in the crystal modifies the buckling height to reduce it. In this work, the measured buckling of 0.09 Å affirms the relaxed state of Bi_ORTH_ in comparison to Bi_TRIG_ (012) [13,15,21]. Almost all studies on Bi_ORTH_ have reported buckling to be present, and the extent of buckling is reported to have a huge influence on the topological properties [10,13,15,21]. According to Lu et al., the measured value of buckling height, i.e., 0.09 Å in this work, places Bi_ORTH_ crystals in a topological regime [13].

Though in-plane lattices are successfully measured, there remains a question of why the diffraction is not observed for conventional high-intensity peaks, i.e., the Bi_ORTH_ $\left(06\bar{2}\right)$ and the Bi_ORTH_ (260), while a low-intensity peak such as the Bi_ORTH_ $\left(15\bar{1}\right)$ is probed successfully. The answer resides in the unique structural arrangement of the Bi_ORTH_ crystal on Si (111) substrates. The 45° in-plane rotation of the Bi_ORTH_ lattice, when projected on Si $\left[1\bar{1}0\right]$ and Si [211] planes, together with the matching out-of-plane lattice, facilitates the formation of a pseudo-cubic sub-lattice, as depicted in Figure 1g–i. To date, RSM-based structural investigations of the Bi_ORTH_ phase have not been reported, which presented certain challenges in the evaluation of diffraction peaks. Nonetheless, based on the values evaluated by the Pythagorean theorem stated in Equations (1)–(3) and the matching out-of-plane lattice parameters, Bi in a pseudo-cubic structure (Bi_CUB_) is estimated to arrange itself with “a” ≈ “b” ≈ “c” ≈ 6.57 Å. Adopting Bi_CUB_ parameters, high-intensity asymmetric peaks are selected, and asymmetric RSMs at the Bi_CUB_ $\left(\bar{2}40\right)$ and the Bi_CUB_ $\left(04\bar{2}\right)$ peaks are successfully acquired, as shown in Figure 8b,c respectively. The difference in the out-of-plane lattices acquired via the Bi_CUB_ $\left(\bar{2}40\right)$ and the Bi_CUB_ $\left(04\bar{2}\right)$ can be linked directly to the Bi_ORTH_$\;(15\bar{1})$ in Figure 8a. In other words, the Bi_ORTH_ and the Bi_CUB_ are two different representations of the same crystal structure. To confirm the lattice symmetry, RSMs at the Bi_CUB_ $\left(\bar{2}40\right)$ and the Bi_CUB_ $\left(04\bar{2}\right)$ peaks are also acquired for 30° and 60° rotated domains, and similar results are attained that can be visualized in Supplementary Figures S5 and S6.

(1) $$a_{CUB}=\sqrt{{a_{ORTH}}^{2}+{c_{ORTH}}^{2}}$$

(2) $$a_{CUB-8\;nm}=\sqrt{4.49^{2}+4.81^{2}}\approx 6.58\;Å$$

(3) $$a_{CUB-25\;nm}=\sqrt{4.57^{2}+4.74^{2}}\approx 6.58\;Å$$

During RSM investigations, it was observed that thickness plays a critical role in the structural arrangement of the Bi_ORTH_ lattice. RSMs acquired at the the Bi_ORTH_$\;(15\bar{1})$ peak in a 25 nm thick epilayer are depicted in Figure 8d, where the in-plane lattice parameters are found to be 4.57 ± 0.02 Å and 4.74 ± 0.02 Å, respectively. It is evident that both in-plane lattices have shifted slightly, and the difference between them (Δd) is reduced from 0.32 Å to 0.17 Å. Moreover, the buckling height is witnessed to enhance three-fold, from 0.09 Å to 0.29 Å, when the layer thickness is increased from 8 nm to 25 nm, indicating the increased strain in the Bi_ORTH_ crystal. A similar effect is also observed by measuring the Bi_CUB_ $\left(\bar{2}40\right)$ and the Bi_CUB_ $\left(04\bar{2}\right)$ peaks depicted in Figure 8e,f, respectively, confirming the enhanced buckling height in the thick Bi_ORTH_ film. An interesting point to mention here is, that, no thickness dependent change in the in-plane lattices of Bi_CUB_ is observed. It is due to the fact that the projected values of both sets of orthorhombic lattices at 8 nm (Figure 8a) and 25 nm (Figure 8d) remain almost identical (Equation (3)). The acquired lattices in a 25 nm-thick Bi_ORTH_ epilayer are found to be in close agreement with earlier reports on Si (111), although the buckling height is still much smaller than the reported value of 0.5 Å [21,26,27,29,41].

## 7. Atomic-Scale Structural Characterization

**Figure 9:**
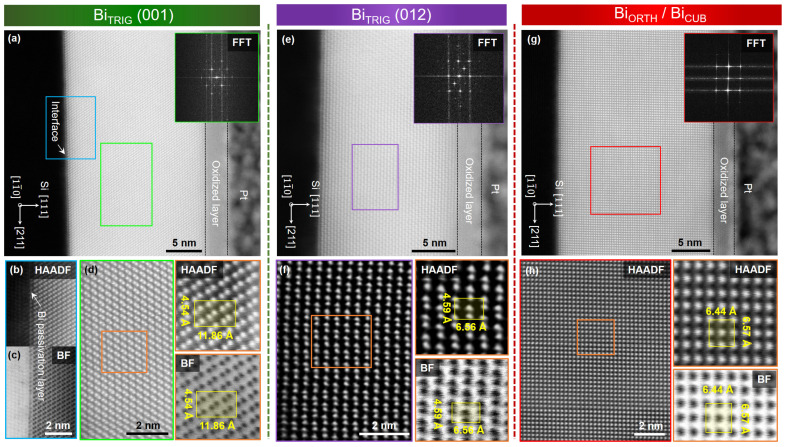
STEM investigations of Bi nanofilm allotropes. (**a**) A cross-sectional HAADF image of Bi_TRIG_ (001) epilayer acquired along Si $\left[1\bar{1}0\right]$ projection. The magnified region at the interface can be visualized via (**b**) HAADF and (**c**) BF images, respectively. The central marked region is further magnified and depicted in (**d**), where the periodic stacking of Bi bilayers can be seen. (**e**) A HAADF image of Bi_TRIG_ (012) grain in a polycrystalline epilayer acquired along Si $\left[1\bar{1}0\right]$ projection. The central marked region is magnified, filtered (band-pass) and depicted in (**f**), where the puckered structures of Bi atoms along with a strong buckling between puckered atoms can be perceived. (**g**) A HAADF image of Bi_ORTH_ (010) epilayer acquired at the cross-section along Si $\left[1\bar{1}0\right]$ projection. The domain is observed to be 2° in-plane rotated with respect to the substrate, which does not allow acquisition of atomically resolved images at the interface. The central highlighted region is magnified and depicted in (**h**), where the pseudo-cubic structure of Bi sub-lattice is evident.

Finally, using scanning transmission electron microscopy (STEM), atomic-scale structural characterizations of Bi nanofilm allotropes are performed. Epilayers from three distinct phases, including the Bi_TRIG_ (001), the Bi_ORTH_ (010), and a polycrystalline phase acquired at T_sub_ = 70 °C containing both the Bi_TRIG_ (001) and the Bi_TRIG_ (012) orientations, are selected. The selected epilayers are then probed via focused ion beam (FIB), and lamellae at the cross-sections of each epilayer along Si $\left[1\bar{1}0\right]$ and Si [211], are extracted. The Bi epilayers, unlike other layered materials, presented serious challenges both in extracting lamellae and in acquiring high-resolution STEM images. On several occasions, the lamellae were observed to be amorphous with a heavily deformed interface at the substrate. A few examples are summarized in Supplementary Figures S7–S10. Such a behavior can be explained by a poor thermal stability of Bi. By carefully fabricating lamellae at low energies in FIB and reducing the exposure time under the focused electron beam during STEM, successful structural investigations are performed, and the key results are summarized in Figure 9.

Figure 9a depicts an overview high-angular annular dark-field (HAADF) image of the Bi_TRIG_ (001) epilayer along the Si $\left[1\bar{1}0\right]$ projection. An atomically sharp interface between Bi_TRIG_ and Si (111) can be visualized in Figure 9b,c, depicting HAADF and bright-field (BF) images, respectively. Before the periodic stacking of Bi bilayers, the appearance of a Bi monolayer passivating the dangling bonds at the Si (111) surface, is evident. It affirms the formation of 1×1—Bi surface, a 2D sheet of Bi, as a seed layer for epitaxial growth of Bi_TRIG_ (001). A magnified HAADF image of the central region, away from the interface, is depicted in Figure 9d, affirming the high structural quality of the epilayer without the appearance of any extended defects.

During the investigation of polycrystalline epilayers, the presence of the Bi_TRIG_ (012) oriented crystals, predicted during the analysis of domain alignment in Figure 7b, is successfully observed. A HAADF image focusing on the Bi_TRIG_ (012) oriented grain is depicted in Figure 9e, while a low-pass filtered, magnified image of a central region is depicted in Figure 9f. It clearly indicates a black-phosphorus-like puckered structure of Bi atoms; however, unlike the black phosphorus, the Bi_TRIG_ (012) exhibits a strong buckling between the Bi atoms that can also be visualized in the image. Atomic distance measurements via line profiles of the HAADF image have revealed the buckling height to vary between 0.43 ± 0.02 Å and 0.48 ± 0.02 Å in a Bi_TRIG_ (012) monolayer. The difference between various measured values at different positions in the acquired image can be linked to localized changes in the tilt angle as the lamella is witnessed to be heavily bent due to the curtain effect imposed by the deposited Pt layer during FIB. Nevertheless, even the smallest measured value of buckling in the Bi_TRIG_ (012) orientation is almost half an order larger than the XRD-measured value of buckling in Bi_ORTH_ thin films, depicted in Figure 8a.

Figure 9g represents a HAADF image acquired at the cross-section of a Bi_ORTH_ epilayer along the Si $\left[1\bar{1}0\right]$ projection. An apparently relaxed and periodic stacking of the Bi monolayer ordered in a cubic-like structure can be witnessed in the image. An atomically sharp and clean interface between the substrate and the Bi_ORTH_ epilayer is also evident. As established during XRD investigations, the unique alignment of the Bi_ORTH_ crystals with a 45° in-plane offset with the Si (111) substrate facilitates the formation of a pseudo-cubic sub-lattice. A magnified HAADF image depicted in Figure 9h represents the Bi_CUB_ structure, with the in-plane and the out-of-plane lattices measured to be 6.57 ± 0.02 Å and 6.44 ± 0.02 Å, respectively. The acquired values from STEM are in good approximation with XRD acquired lattices, depicted in Figure 8d–f.

## 8. Thickness-Dependent Phase Transformations

With such a thorough study of Bi epitaxial growth, most of the earlier introduced obscurities regarding nanofilm allotropes are addressed. However, one issue still remains unaddressed: the thickness-dependent phase transformation in Bi epilayers. As mentioned earlier, various reports have confirmed the existence of Bi_ORTH_ phase during early stages of growth where the Bi_ORTH_ phase is identified in the form of crystallites not only on Si (111) but also on Al_2_O_3_ (0001) and HOPG substrates [10,13,15,16,27,29,39]. According to these reports, the Bi_ORTH_ phase is observed to be stable in ultra-thin films consisting of up to four monolayers (4ML), approx. 1.3 nm thick. As soon as layer thickness increases, a transformation from Bi_ORTH_ into Bi_TRIG_ (001) phase is reported to initiate, which remains true if the layer thickness remains below 8–10 ML (approx. 3.5 nm). However, any film having a thickness > 3.5 nm is reported to crystallize only in the Bi_TRIG_ (001) phase. These observations are often supported by theoretical modelling and simulations [21,29,41]. The findings in this work mostly contradict the above-mentioned reports, as the epitaxial growths of the Bi_TRIG_ (001) and the Bi_ORTH_ phases are performed independently of each other.

**Figure 10:**
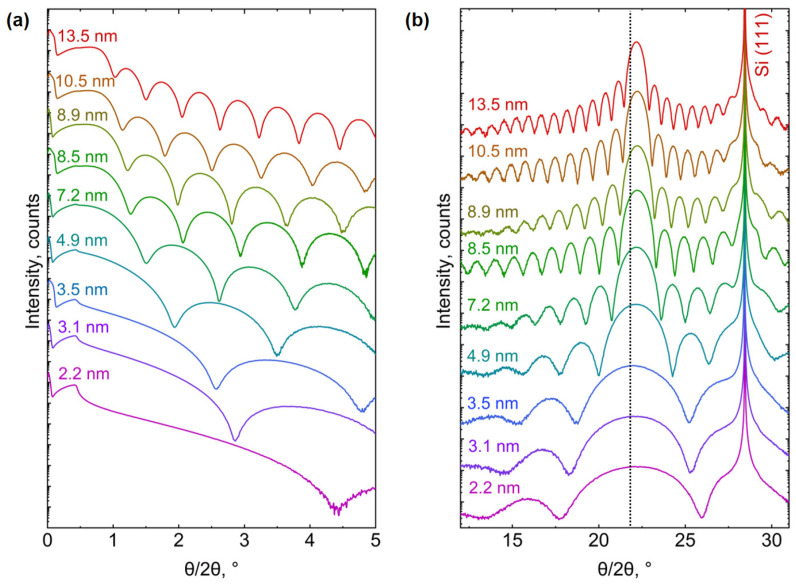
Thickness series of the Bi_TRIG_ (001) phase. (**a**) XRR-measured curves of epilayers ranging in thickness from 2.2 nm to 13.5 nm, whereas (**b**) depicts the corresponding diffraction patterns. The dotted black line in (**b**) indicates a slight shift in the out-of-plane lattice parameter due to thin-film effect.

**Figure 11:**
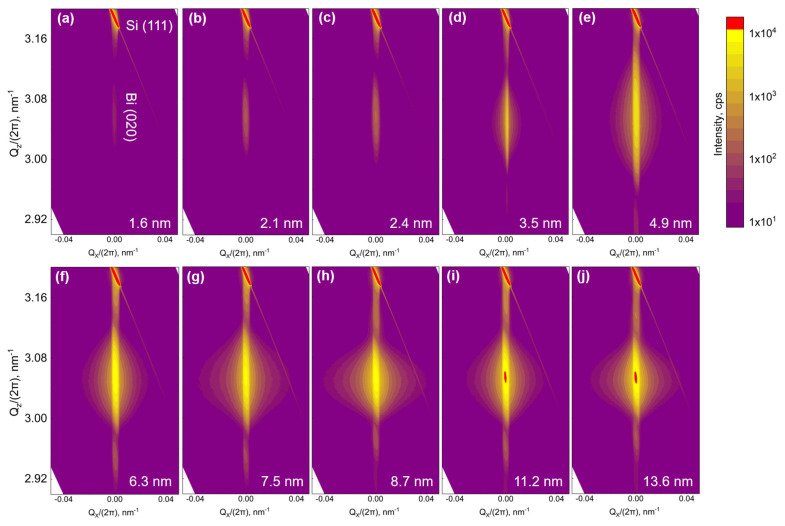
Thickness series of the Bi_ORTH_ (010) phase. The presence of a high-intensity Si (111) peak in vicinity of the Bi_ORTH_ (020) peak does not facilitate the analysis of thin films via diffraction pattern; however, it can be adopted as a reference in a symmetric RSM. Various symmetric RSMs of Bi_ORTH_ epilayers are acquired at the (020) peak, while the epilayer thickness is varied from (**a**) 1.6 nm to (**b**) 2.1 nm, (**c**) 2.4 nm, (**d**) 3.5 nm, (**e**) 4.9 nm, (**f**) 6.3 nm, (**g**) 7.5 nm, (**h**) 8.7 nm, (**i**) 11.2 nm and (**j**) 13.6 nm.

However, before releasing a final statement, the dependency of phase transformation on the layer thickness is investigated, and the thickness-dependent controlled epitaxy of both phases, i.e., the Bi_TRIG_ (001) and the Bi_ORTH_ (010), is performed in a systematic manner. The growth parameters are adjusted to the optimum values for the Bi_TRIG_ (001), (T_sub_, R_TF_) = (40 °C, 22 nm/h), and epilayers ranging in thickness from 2.2 nm to 13.5 nm are prepared. Figure 10 represents the XRD characterization of the Bi_TRIG_ (001) thickness series, where (a) depicts XRR curves, and (b) depicts the corresponding diffraction patterns. It is evident that all epilayers exhibit only the Bi_TRIG_ (001) phase, and no indication of phase duality is observed. The black-dotted line highlights a shift in the out-of-plane lattice constant due to the scaling effect. This effect has already been reported for GeTe [42]; however, it is not discussed here for Bi. As the focus of this work is on the thickness-dependent phase transformation, the investigation of the scaling effect within the same phase is outside the scope of this work. Nonetheless, the growth parameters are then adjusted to the Bi_ORTH_ optimum values, (T_sub_, R_TF_) = (100 °C, 32 nm/h), and epilayers in thickness ranging from 1.6 nm to 13.6 nm are prepared. The results are summarized via symmetric RSMs around the Bi_ORTH_ (020) peak and are depicted in Figure 11.

The presence of the Bi_ORTH_ $\left(020\right)$ peak in layers having a thickness >> 3.5 nm is clear evidence that the Bi_ORTH_ epilayers can be grown phase-segregated and that layer thickness has no direct relation to phase transformation. Still, it is of critical importance to identify probable factors that may have led to the thickness-dependent phase transformation of Bi_ORTH_ in earlier-reported studies. The two key differences in this work compared to earlier reports are the substrate surface and the selection of growth parameters. (1) In this work, all growths are performed on Si $\left(111\right)$ 1×1 conventional surfaces passivated by Bi-monolayers, whereas all earlier reports have adopted Si (111) $\sqrt{3}\times \sqrt{3}-\beta$ or 7×7 reconstructed surfaces that may have influenced the starting phase of Bi films. (2) For the first time, phase-dependent optimum growth parameters are evaluated and implemented in this work to affirm the phase purity of the growing epilayer The thickness-dependent phase transformation may be realized if similar growths are carried out with non-optimal parameters, i.e., in the “transformation zone” or at the zone boundary. However, it is just speculation until confirmed.

**Figure 12:**
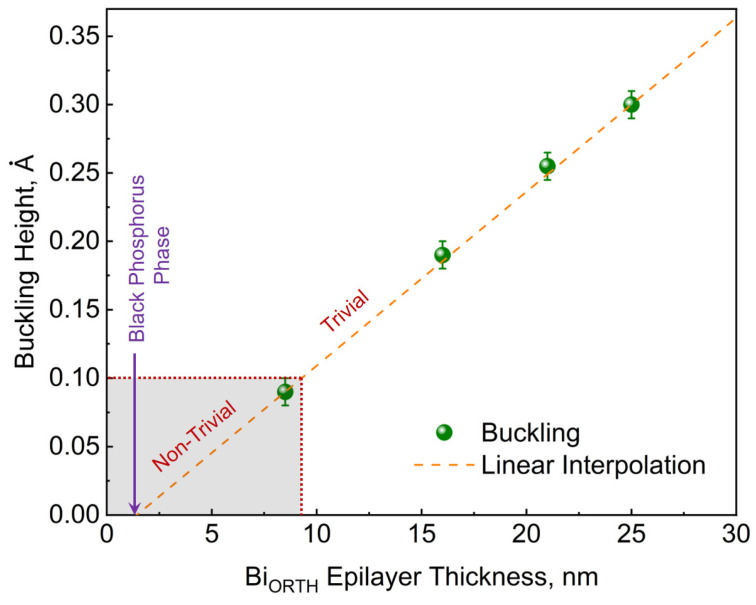
Buckling height vs. topology in the Bi_ORTH_ epilayers. Linear interpolation of thickness-dependent acquired values indicate that the buckling height in Bi_ORTH_ epilayers should reach zero, which would allow the realization of the black-phosphorus-like phase of Bi (Bi_BP_). According to the theoretical model in [13], any Bi_ORTH_ epilayer hosting the buckling height ≤ 0.1 Å must exhibit topology, and thus, all Bi_ORTH_ epilayers with thickness ≤ 9 nm are predicted to exhibit non-trivial attributes.

Though a thickness-dependent phase transformation in the Bi_ORTH_ epilayers is not realized, a continuous shift in the lattice parameters is observed. Figure 11 indicates only a slight change in the out-of-plane lattice depending on the epilayer thickness, whereas major changes are perceived in the in-plane structure of Bi_ORTH_. It is observed that at low thicknesses, epilayers exhibit a relatively large difference between the in-plane lattice parameters, while the buckling height remains rather small, as witnessed in Figure 8a. With increasing thickness, the difference between in-plane lattice parameters decreases, whereas the buckling height increases, thus affirming the increasing strain in the epilayer, as depicted in Figure 8d. The observed trend of buckling height dependency on the Bi_ORTH_ layer thickness is summarized in Figure 12. Using linear interpolation, it is identified that the black-phosphorus phase of Bi (Bi_BP_) that exhibits zero-buckling can only be realized if the epilayer thickness remains below 1.32 nm. As the thinnest Bi_ORTH_ epilayer achieved in this work is only 1.63 nm thick, the requirements for realization of the Bi_BP_ phase are not met, and thus, only the Bi_ORTH_ phase is observed. The measured buckling values in Figure 12 also indicate that if such an ultra-thin epilayer is achieved, the probability of realizing the Bi_BP_ phase is high.

Furthermore, the buckling height is reported to have a huge influence on the topological properties of Bi_ORTH_. According to Lu et al., all epilayers with buckling height ≤ 0.1 Å exhibit non-trivial topology, whereas increasing buckling height transforms the epilayer into a trivial phase [13]. Based on this, all Bi_ORTH_ epilayers prepared in this work having a thickness ≤ 9 nm must exhibit non-trivial attributes, which was only true for approx. 1.3 nm thick epilayers in all earlier-reported studies. It facilitates the realization of Bi_ORTH_-based quantum devices, as the achievement of fully coalesced 9 nm-thick epilayers is far less complex than 1.3 nm. The increasing buckling height in the epilayer indicates that after a certain thickness, the Bi_ORTH_ epilayer becomes unfavorable and will cross the limit of quantum confinement and transform into the Bi_TRIG_ (001) phase, just as reported in earlier studies. This hypothesis is confirmed with an attempt to grow a 45 nm-thick Bi_ORTH_ epilayer, which resulted in a polycrystalline film containing the Bi_ORTH_ and the Bi_TRIG_ (001) structures, confirming that the maximum stable thickness of the Bi_ORTH_ phase is somewhere between 30 nm (depicted in Figure 9) and 45 nm.

The groundwork for manufacturing nano-devices has now been laid with the successful phase-selective epitaxy of Bi-thin films. However, one must proceed with the utmost caution, as Bi oxidizes quickly when exposed to air. In the initial stages of oxidation, Bi forms a polycrystalline BiO_x_ layer, as depicted in Figure 9e. The polycrystalline nature of BiO_x_ makes it significantly challenging to remove during metallization, and access to a pristine epi-surface to realize atomically clean and sharp interface remains a challenge. A detailed and systematic study of surface oxidation in Bi thin films is performed; however, it is not discussed here as it is outside the scope of this work. Furthermore, due to a low thermal stability, conventional fabrication techniques may degrade the crystal quality of Bi epilayers, which makes it even more challenging to realize functional quantum devices. An earlier-proposed platform with an on-chip stencil mask provides a way around the fabrication challenges [43], whereas the issue of surface oxidation can be addressed via in situ capping of the epitaxial surface with a passivation layer. Various topological insulator thin films are reported to have successfully achieved surface passivation using an electron beam-deposited thin film of stoichiometric Al_2_O_3_ [43,44,45,46]. In some circumstances, a 2 nm thermally evaporated film of elemental Al is adopted as passivation [47,48,49,50,51,52]. Al readily oxidizes and transforms into AlO_x_ when exposed to air, protecting the underlying surface from deterioration and aging. By combining phase-selective growth of Bi with an on-chip stencil mask [43] and in situ surface passivation, this study can be used to realize promising quantum devices for a variety of novel topological and neuromorphic applications.

## 9. Conclusions

In conclusion, we have demonstrated the high-quality growth of Bi thin films of various phases via molecular beam epitaxy on Si (111) 1×1 surfaces. First, the growth parameters for the trigonal phase, i.e., Bi_TRIG_ (001), were optimized to T_sub_ = 40 °C and R_TF_ = 22 nm/h, yielding rocking curves as sharp as 53 ± 10″. By increasing the substrate temperature from T_sub_ = 40 °C to 95 °C, a structural transformation of the Bi epilayers into the orthorhombic phase Bi_ORTH_ (010) was observed. After optimizing the crystal quality of the Bi_ORTH_ (010) to a rocking curve value of 58 ± 10″, φ-scans of various growth series were performed to cast light on the dynamic transformation between the trigonal and orthorhombic Bi phases as a function of T_sub_ and R_TF_. Strikingly, it was found that the six-fold symmetry of the twinned trigonal phase, i.e., Bi_TRIG_ (001), transforms partly into an intermediate phase before fully relaxing into the orthorhombic phase. The observed intermediate phase with its characteristic three-pointed peaks was identified as the Bi_TRIG_ (012) phase. As the out-of-plane lattices of Bi_ORTH_ (010) from Bi_TRIG_ (012) orientations are very similar, asymmetric RSMs were used to distinguish the intermediate from the orthorhombic phase. Next, the amount of buckling in phase-pure Bi_ORTH_ (010) thin films was investigated, as it was proposed that the degree of buckling determines the topological class the Bi crystal belongs to. It was found that the buckling height increases with the thickness of the epilayers. Unlike earlier reports, the maximum height of phase-pure Bi_ORTH_ (010) before transforming into Bi_TRIG_ (001) was found to be 30 nm, in contrast to the 1.3 nm value reported previously as the critical height. The enhanced maximum height observed in our study allows for the exploration of the topological phase in Bi_ORTH_ (010), which has been predicted to be present in films as thick as 9 nm. We attribute this key finding to the selection of the Si (111) 1×1 surface that we use for Bi epitaxy. Earlier studies used either Si (111) 7×7 or $\sqrt{3}\times \sqrt{3}-\beta$ reconstructions, which only allowed for the growth of non-coalesced crystallites of Bi_ORTH_ (010), which are not suitable for systematic integration into device circuitry. The 1×1 surface used here, however, allows for the phase-pure and thickness-independent growth of fully coalesced thin films of high-quality Bi_ORTH_ (010) up to 30 nm. These results pave the way to study and exploit the topological phases and phase transitions in Bi_ORTH_ (010) in future quantum devices.

## Data Availability

The data presented in this study are openly available in JülichDATA at https://doi.org/10.26165/JUELICH-DATA/B2NTJY.

## Supplementary Materials

The following supporting information can be downloaded at: https://www.mdpi.com/article/10.3390/nano13142143/s1, Figure S1. XRD investigation of Bi_TRIG_ (012) epilayer via asymmetric RSMs probed at (a) (116), (b) (030), (c) $\left(\bar{3}18\right)$ and (d) $\left(\bar{3}\bar{1}4\right)$ peaks. Figure S2. XRD diffraction peaks map of Bi_ORTH_ (010) at φ = 45°. Figure S3. XRD diffraction peaks map of Bi_CUB_ along Si $\left[1\bar{1}0\right]$ projections. Figure S4. XRD diffraction peaks map of Bi_CUB_ along Si [211] projection. Figure S5. Evaluation of lattice parameters via measuring RSMs at Bi_CUB_ $\left(\bar{2}40\right)$ and Bi_CUB_ $\left(04\bar{2}\right)$ peaks are 30° rotated domain. Figure S6. Evaluation of lattice parameters via measuring RSMs at Bi_CUB_ $\left(\bar{2}40\right)$ and Bi_CUB_ $\left(04\bar{2}\right)$ peaks are 60° rotated domain. Figure S7. HAADF images of Bi_TRIG_ (001), Bi_TRIG_ (012) and Bi_ORTH_ (010) epilayers confirming the large grain sizes and high crystal quality of the epilayers. Figure S8. HAADF images of Bi_TRIG_ (001) epilayers along Si [211] projection. Figure S9. HAADF and bright field images indicating the effect of image acquisition time on the Bi_TRIG_ (001) epilayers. The average acquisition time in (b) is higher than (a). The damaged regions in (b) are the confirmation of Bi instability under elongated exposure. Figure S10. Sets of HAADF and bright field images representing the continually increasing structural damage of Bi epilayer with increasing illumination time under the electron beam. The interface between Bi and Si is witnessed to be the most affected region. The acquisition time in all images remains similar.
