# Supplementary material for: Phase-Selective Epitaxy of Trigonal and Orthorhombic Bismuth Thin Films on Si (111)
Source: Nanomaterials (Basel). 2023 Jul 24;13(14):2143. doi: 10.3390/nano13142143 (PMC10386495; doi:10.3390/nano13142143)
Supplement: Supplementary file 1 [file nanomaterials-13-02143-s001.zip › nanomaterials-2500996-supplementary.pdf]

nanomaterials

1. Methodology

- **Substrate:** 4-inch Si (111) wafers having the resistivity > 5000 ohm.cm and a miscut angle of 0.05° are diced into 10 mm x 10 mm pieces. Each piece of the substrate before epitaxy is chemically cleaned via Piranha 1:1 solution, a mixture of 37% hydrogen peroxide (H_2O_2) and 96% Sulfuric acid (H_2SO_4), for 10 minutes and rinsed with the de-ionized water for 15 minutes. Before loading the substrate into the MBE chamber, the native oxide on the Si (111) surface is etched via 1% HF solution for 5 minutes and later rinsed with the de-ionized water for a couple of minutes to make sure that the dangling bonds are saturated with H^+ ions.
- **Growth:** The epitaxial growth is perform in an ultra-high vacuum MBE chamber. The chamber has the base pressure of $1x10^{-10}$ mbar which drops to approx. $5x10^{-10}$ mbar during the growth. The substrates are annealed in the growth chamber at 600 °C for 15 mins to make sure the 1x1 surfaces do not reconstruct and the Si dangling bonds are re-exposed (H^+ removed), before it is cooled down to the growth temperature. At the same time, Bi (purity 99.9999 %) in the Knudsen cell is heated with temperature range from 480 °C to 575 °C to stabilize the Bi flux, in order to achieve the target growth rate (R_{TF}). The thin films are grown with a duration of 30 min to 90 mins depending upon the applied R_{TF}.
- **Surface Morphology:** Scanning electron microscopy (SEM) is performed via "Leo 1550 ZAT" setup using an acceleration voltage between 2 to 5 KeV, which provided the information about the surface texture.
- **Structural Analysis:** The crystal structure features of thin films are characterized by X-ray diffraction (XRD) via "Rigaku Smartlab" system utilizing a Cu Kα_1 radiation source (λ = 1.540562 Å) with a rotating anode. The lamellae for TEM are prepared by a focused ion beam (FIB) system "FEI Helios Nanolab 600i". Before cutting with the Ga ions in FIB, Pt layers were deposited on the sample surface to protect the specimen from damaging. Plasma cleaning was carried out to remove any surface contamination of the lamella before inserting into the microscope. Scanning transmission electron microscopy (STEM) is performed with "FEI Titan G2 80-200 ChemiSTEM" microscope, which is equipped with a spherical aberration probe "Cs" corrector and the annular dark-field detectors. High-angular annular dark-field (HAADF) and Bright filed (BF) images are acquired at 200 kV.

2. Identification of Bi_{TRIG} (012) phase via RSM

The identification of Bi_{TRIG} (012) phase is conducted via measuring various asymmetric RSMs. The possible peak map can be seen in Figure S1 and S2; whereas, a few examples of measured map can be seen in Figure S3.

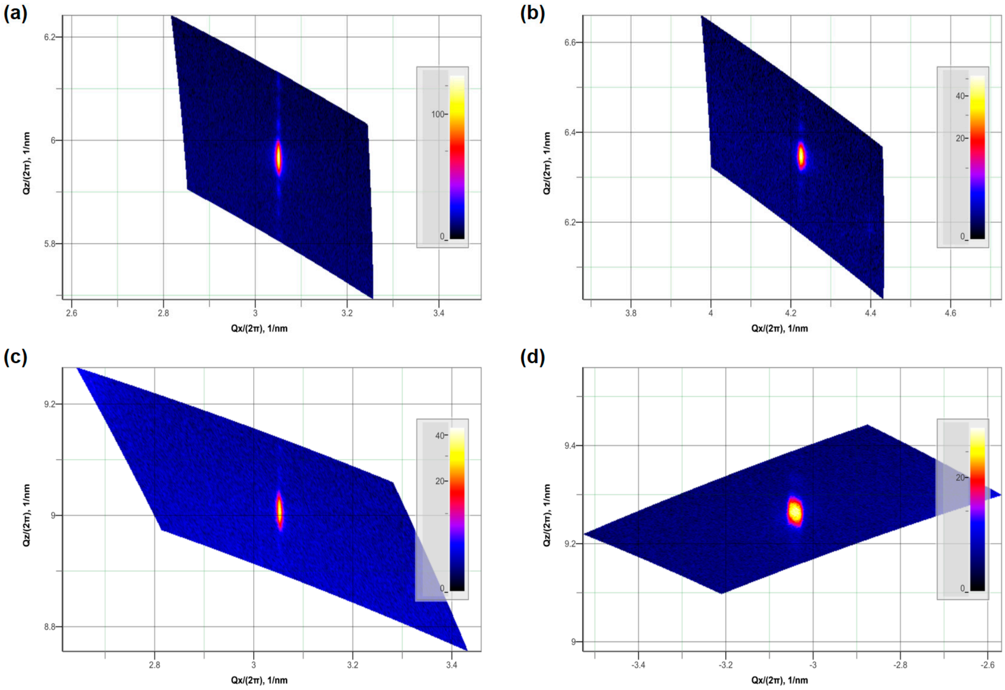

Figure S1. XRD investigation of BiTRIG (012) epilayer via asymmetric RSMs probed at (a) (116), (b) (030), (c) $(\bar{3}18)$and (d) $(\bar{3}\bar{1}4)$ peaks.

3. XRD characterization of BiORTH epilayers

The diffraction peak map of BiORTH (010) along 45° rotated surface along with BiCUB peaks map along Si $[1\bar{1}0]$ and [211] projections are depicted in Figure S2-S4 respectively.

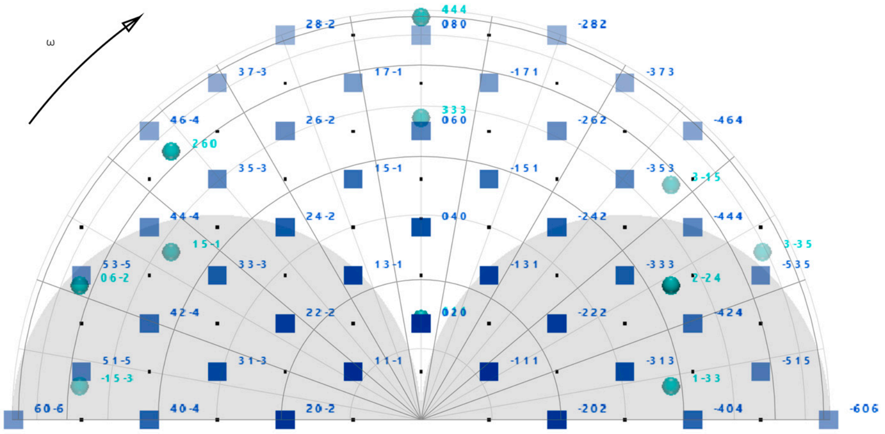

Figure S2. XRD diffraction peaks map of BiORTH (010) at φ = 45°.

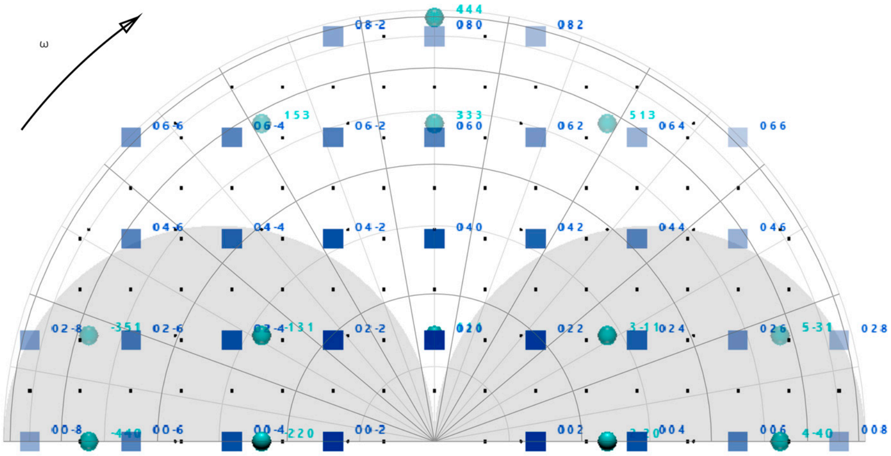

Figure S3. XRD diffraction peaks map of Bicub along Si [$1\bar{1}0$] projections.

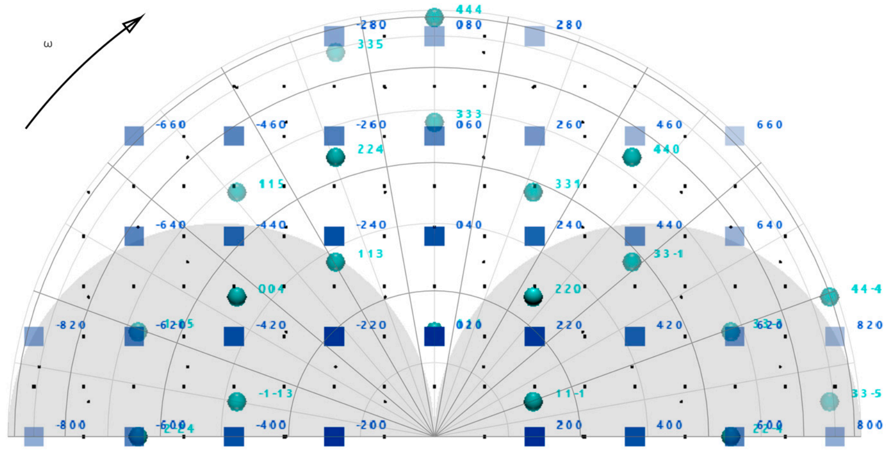

Figure S4. XRD diffraction peaks map of Bicub along Si [211] projection.

4. RSM of Biorth epilayer along 30° and 60° rotational domains

To confirm the lattice symmetry of BiCUB phase, RSMs at BiCUB $(\bar{2}40)$ and BiCUB $(04\bar{2})$ peaks are acquired for 30° and 60° rotated domains and depicted in Figure S5 and S6 respectively.

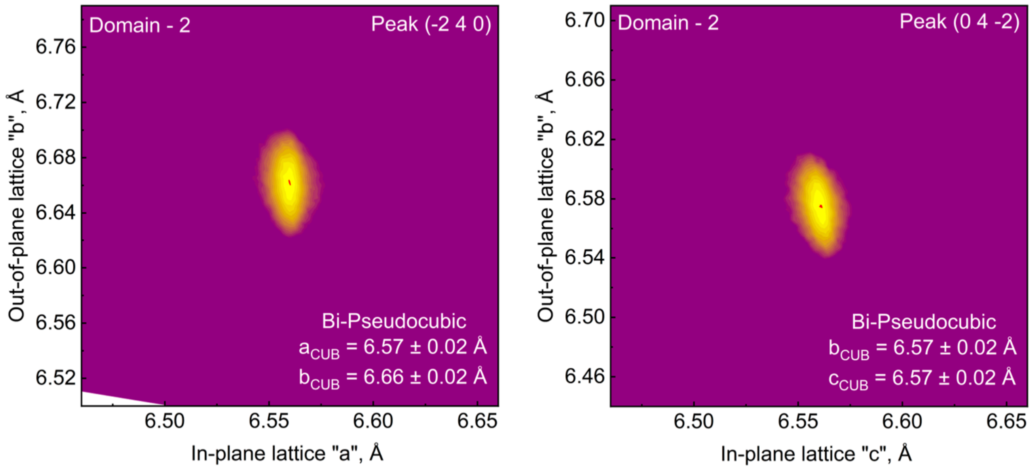

Figure S5. Evaluation of lattice parameters via measuring RSMs at BiCUB $(\bar{2}40)$ and BiCUB $(04\bar{2})$ peaks are 30° rotated domain.

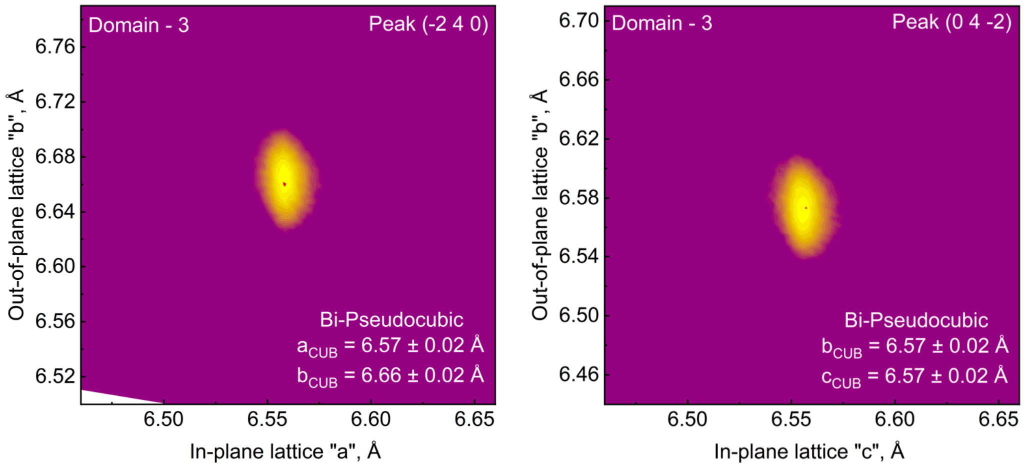

Figure S6. Evaluation of lattice parameters via measuring RSMs at BiCUB $(\bar{2}40)$ and BiCUB $(04\bar{2})$ peaks are 60° rotated domain.

5. STEM investigations

STEM investigation has provided in-detailed structural information of Bi nanofilm allotropes. Figure S7 depicts HAADF images representing the large grain sizes of Bi_{TRIG} (001), Bi_{TRIG} (012) and Bi_{ORTH} (010) epilayers. Figure S8 represents a couple of cross-sectional HAADF images of Bi_{TRIG} (001) epilayer along Si [211] projection. These investigations were performed after a series of failure due to thermal instability of Bi. A couple of examples of Bi instability under electron beam with elongated exposure are depicted in Figure S9 and S10.

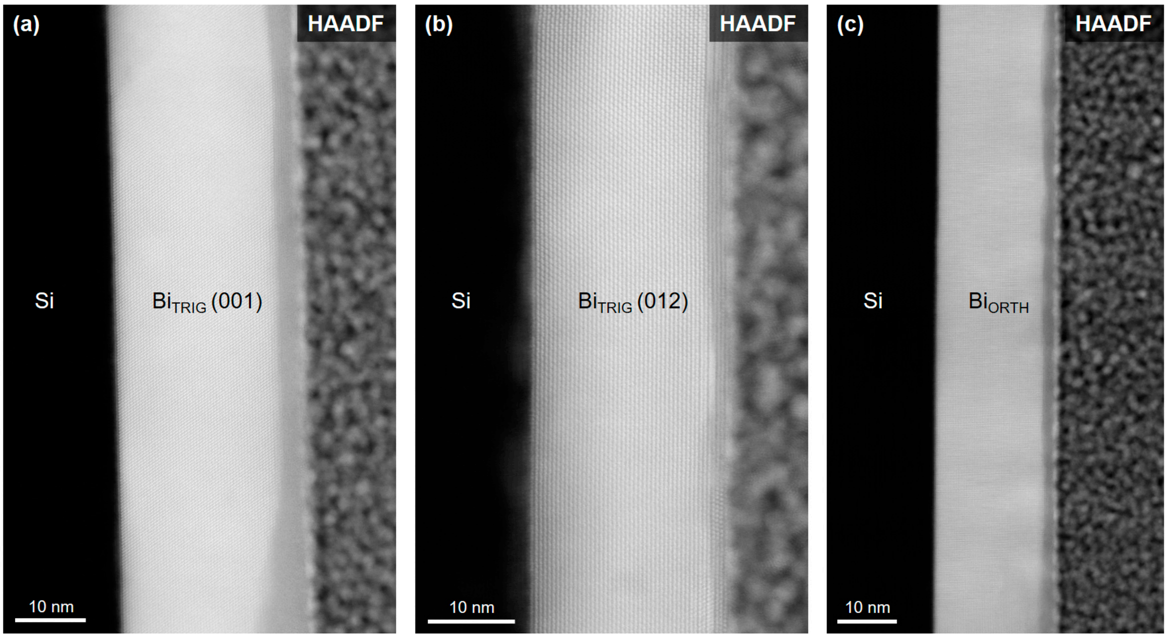

Figure S7. HAADF images of Bi_{TRIG} (001), Bi_{TRIG} (012) and Bi_{ORTH} (010) epilayers confirming the large grain sizes and high crystal quality of the epilayers.

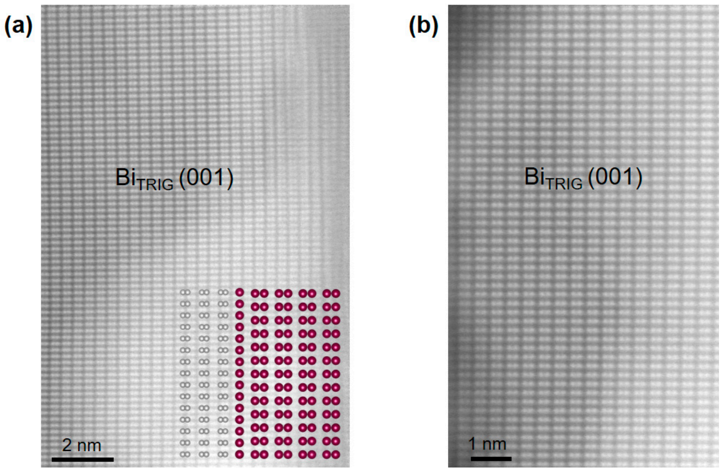

Figure S8. HAADF images of Bi_{TRIG} (001) epilayers along Si [211] projection.

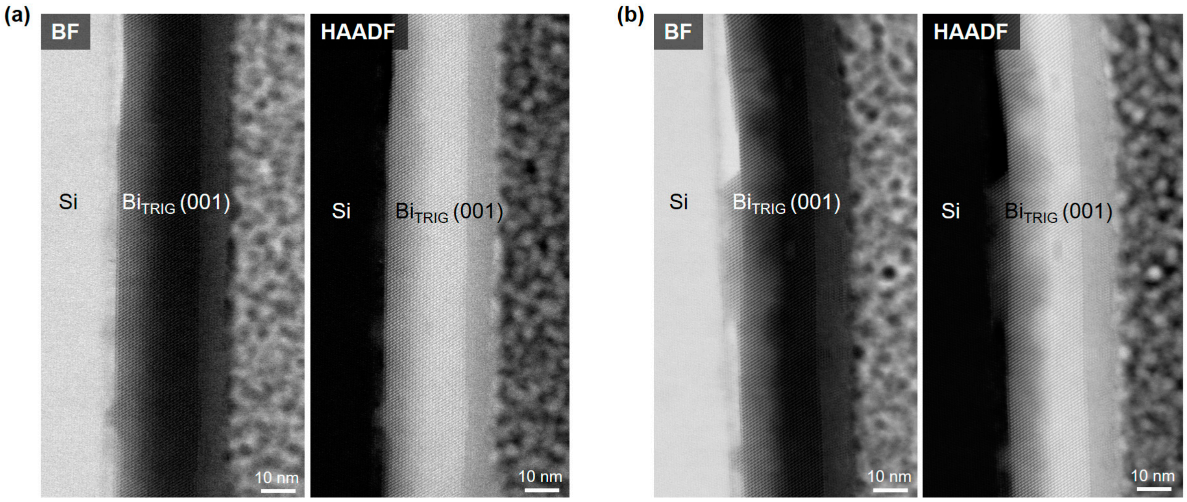

Figure S9. HAADF and bright field images indicating the effect of image acquisition time on the BiTRIG (001) epilayers. The average acquisition time in (b) is higher than (a). The damaged regions in (b) are the confirmation of Bi instability under elongated exposure.

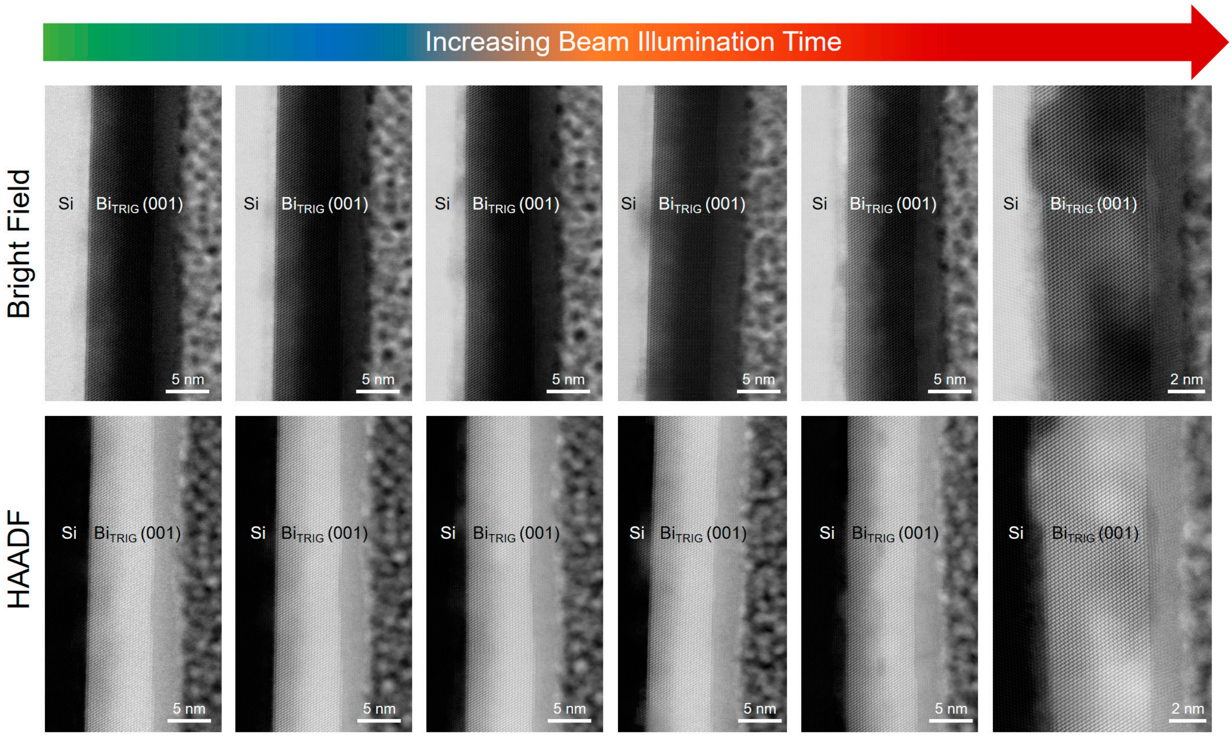

Figure S10. Sets of HAADF and bright field images representing the continually increasing structural damage of Bi epilayer with increasing illumination time under the electron beam. The interface between Bi and Si is witnessed to be the most affected region. The acquisition time in all images remains similar.
